# Assessment of antibiotic susceptibility in *Lactobacillus* isolates from chickens

**DOI:** 10.1186/s13099-017-0203-z

**Published:** 2017-09-19

**Authors:** Marta Dec, Renata Urban-Chmiel, Dagmara Stępień-Pyśniak, Andrzej Wernicki

**Affiliations:** Sub-Department of Veterinary Prevention and Avian Diseases, Institute of Biological Bases of Animal Diseases, Faculty of Veterinary Medicine, University of Life Sciences in Lublin, Akademicka 12, 20-033 Lublin, Poland

**Keywords:** Antibiotic susceptibility, *Lactobacillus*, Poultry, Resistance genes

## Abstract

**Background:**

The aim of this study was to determine the susceptibility of 88 *Lactobacillus* isolates derived from chickens to antibiotic substances and to detect drug-resistance genes.

**Results:**

The minimal inhibitory concentration of 13 antimicrobial substances was determined by the broth microdilution method, and resistance genes were detected by PCR. We recorded a high prevalence of resistance to tiamulin (90% resistant isolates), tetracyclines (74%) and lincomycin (70%), and a moderately high frequency of resistance to enrofloxacin (48%), macrolides (42%), aminoglycosides (12.5–31%), ampicillin (26%) and chloramphenicol (23%). Multi-drug resistance was observed in 79.5% of isolates. The presence of resistance genes was generally correlated with phenotypic resistance, but some molecular determinants were also recorded in susceptible isolates. Among tetracycline resistance genes, the most frequently identified was *tetW* (45% isolates), followed by *tetM* (26%) and *tetL* (24%). The *ermB*, *ermC* and *lnuA* genes, associated with resistance to macrolides and lincosamides, were observed in 39, 12 and 39% of isolates, respectively. Among genes determining resistance to aminoglycoside antibiotics, we identified *ant(6)*-*Ia* (10% of isolates), *aac(6*′*)*-*Ie*-*aph(2*′*)*-*Ia* (8%), *aph(2*″*)*-*Ic* (6%) and *aadE* (4.5%). The *cat* gene was present in 32 isolates, including 8 of 20 found to be resistant to chloramphenicol. Two genes encoding efflux pumps were identified—the *acrA* gene was present in all isolates tested, and 10 of 79 lactobacilli determined to be phenotypically resistant to tiamulin contained the *lsaE* gene. We were unable to explain the resistance mechanism of *Lactobacillus* isolates to ampicillin, but showed that it did not involve the production of β-lactamases.

**Conclusions:**

Our findings indicate that intestinal lactobacilli should be considered a reservoir of resistance genes and that antibiotics must be used prudently in poultry production. The data derived from this study can be used as a basis for reviewing current microbiological breakpoints for categorization of susceptible and resistant strains within the genus *Lactobacillus*.

## Background

Lactobacilli are Gram-positive rod-shaped non-pathogenic bacteria considered to be beneficial components of the gastrointestinal microbiota of humans and animals, including birds. They play an important role in the physiology of their host, as they maintain the microbial balance around mucous membranes via ecological interactions with the resident flora and beneficially influence the immune system via the GALT. Moreover, they improve digestion and assimilation of nutrients and remove toxic substances [1]. Owing to their health-promoting properties, many *Lactobacillus* strains are used to produce probiotic preparations for humans and animals, and interest in applications for these bacteria continues to grow.

As modern intensive poultry farming and the associated high density of birds are conducive to the rapid spread of germs, the demand for veterinary drugs, including antibiotics, is high. However, antibiotics are becoming less effective due to bacterial resistance, and the hypothesis that the gastrointestinal tract (GIT) acts as a reservoir of antibiotic resistance genes is widely accepted [2]. High density of micro-organisms within this ecosystem facilitates the exchange of resistance genes among members of the microbiota, including both commensals and pathogens. Antibiotic-resistant strains not only pose a danger to animals, but as they spread via the food chain, also contribute to problems in humans.

The aim of this study was to determine the susceptibility of *Lactobacillus* strains derived from chickens to antibiotic substances and to detect drug-resistance genes. To the best of our knowledge, the current literature does not provide comprehensive studies on the antibiotic susceptibility of chicken lactobacilli. There are only two reliable reports [3, 4] of phenotypic and genotypic susceptibility of chicken lactobacilli to tetracycline and MLS antibiotics (macrolides, lincosamides and streptogramins).

Most of the antibiotics and chemotherapeutics tested are currently approved for treatment of poultry diseases in the EU. Tetracyclines, such as oxytetracycline, chlortetracycline, and doxycycline, are recommended for controlling mycoplasmosis and chlamydiosis in poultry, as well as against susceptible strains of various bacteria, including spirochetes, and some protozoa. Macrolides and lincosamides are common therapeutic agents for necrotic enteritis (*Clostridium perfringens*) and intestinal spirochetes (*Brachyspira pilosicoli*), while tylosin and tiamulin are important drugs in controlling chronic respiratory diseases caused by mycoplasmas [5]. Aminoglycosides are particularly effective against Gram-negative bacteria and are poorly absorbed from the gastrointestinal tract. Hence neomycin is commonly used against enteric infections (e.g., salmonellosis or colibacillosis) in poultry [5]. Penicillins, such as amoxicillin, are effective against susceptible strains of various Gram-positive and Gram-negative bacteria, especially in the treatment of *E. coli* septicaemia, salmonellosis, pasteurellosis, necrotic enteritis and chronic respiratory disease in poultry. Fluoroquinolones, including enrofloxacin, flumequine and difloxacin, are synthetic broad-spectrum bactericidal drugs that are frequently used in poultry production to treat salmonellosis, colibacillosis and fowl cholera [6].

The mechanism of action of tetracyclines, MLS antibiotics, aminoglycosides, pleuromutilins and chloramphenicol is based on inhibition of protein synthesis in bacterial cells. Tetracyclines bind to the 30S subunit of ribosomes and prevent the attachment of tRNAs carrying amino acids. MLS antibiotics, tiamulin, and chloramphenicol bind to the 50S ribosomal subunit and/or to peptidyl transferase—an enzyme responsible for forming peptide bonds between amino acids [7]. Beta-lactam antibiotics inhibit bacterial cell wall synthesis by interacting with penicillin binding proteins (PBPs) [5], while fluoroquinolones inhibit DNA replication by binding to the DNA gyrase [6].

The use of antibacterial agents creates selective pressure for the emergence of resistant strains, both pathogenic and commensal. Antibiotic resistance, which is implicated in elevated morbidity and mortality rates as well as increased treatment costs, is considered a major global public health threat (http://www.who.int/drugresistance/en/). Bacteria may be intrinsically resistant to antimicrobial agents or may acquire resistance by de novo mutation or via the acquisition of resistance genes from other organisms. Acquired resistance genes may enable a bacterium to produce enzymes that destroy the antibacterial drug, to express efflux systems that prevent the drug from reaching its intracellular target, to modify the drug’s target site, or to produce an alternative metabolic pathway that bypasses the action of the drug. Many antibiotic resistance genes are carried on plasmids, transposons or integrons that can act as vectors that transfer these genes to other members of the same bacterial species, as well as to bacteria of other genera or species. Horizontal gene transfer may occur via three main mechanisms: transformation, transduction or conjugation [8].

Lactobacilli are commonly used as probiotics and according to the EFSA’s FEEDAP Panel (European Food Safety Authority Panel on Additives and Products or Substances used in Animal Feed) all bacterial strains intended for use as feed additives must be examined to establish their susceptibility to the most relevant antibiotics and chemiotherapeutics. As a basic requirement, the minimum inhibitory concentration (MIC) of the antimicrobials should be determined in order to distinguish susceptible and resistant strains. Strains carrying acquired resistance should not be used as a feed additives unless it can be demonstrated that it is a result of chromosomal mutation(s) [9].

## Methods

### Bacterial isolates

A total of 88 *Lactobacillus* isolates from fresh faeces or cloacae of 30 healthy chickens. Samples were collected from 2012 through 2013 from eight large-scale poultry farms (six broiler farms and two raising Green-legged Partridge hens) located in different parts of Poland, with three or four from each farm. The birds were from 2 to 49 days old. The number of *Lactobacillus* isolates derived from individual farms ranged from 7 to 17. The bacterial isolates had previously been identified to the species level using MALDI-TOF mass spectrometry and Amplified 16S Ribosomal DNA Restriction Analysis (16S-ARDRA) [10]. Lactobacilli belonged to the species *L. salivarius* (n = 31), *L. johnsonii* (n = 21), *L. crispatus* (n = 12), *L. reuteri* (n = 10), *L. ingluviei* (n = 8), *L. saerimneri* (n = 3) and *L. agilis* (n = 3).

The strains were stored in deMan Rogosa Sharpe broth (MRS, BTL, Poland) containing 20% glycerol at −80 °C. Prior to antimicrobial susceptibility testing the cultures were streaked on lactic acid bacteria (LAB) susceptibility test medium (LSM) [11] and incubated overnight at 37 °C in 5% CO_2_.

### Determination of minimal inhibitory concentration

Antibiotic susceptibility of all bacterial isolates was determined by the broth microdilution procedure [12], using the LSM medium recommended by the International Organization of Standardization (ISO)/International Dairy Federation (IDF) [13].

All antimicrobial agent powders were obtained from Sigma-Aldrich (Poland). Enrofloxacin (Enrocin, 50 mg/ml) was purchased from Vet-Agro (Poland) and tiamulin (Biomutin, 200 mg/ml) from BIOWET DRWALEW S.A. (Poland).

Inocula were prepared by suspending bacteria in 0.9% NaCl so that the optical density (OD) of the suspension at 600 nm was 0.5. Microdilution plates were inoculated with 50-μl of a 1:500-diluted (in LSM broth) inoculum and 50 μl of the appropriate antibiotic concentration (stock solution previously dissolved in LSM), resulting in the final range of concentrations shown in Table 1. After the plates were incubated at 37 °C in 5% CO_2_ for 48 h, MIC values were read as the lowest concentration of an antimicrobial agent at which visible growth was inhibited.

**Table 1:** Antimicrobial substances and their dilutions used to determine MICs

Antimicrobial substance	Group of antimicrobial agent	Diluent to prepare stock solution	Range of concentration (µg/ml)
Ampicilin	β-Lactams	BR II^a^	0.125–64
Tetracycline	Tetracyclines	0.1 M HCl	1–512
Doxycycline			0.5–256
Erythromycin	Macrolides	Methanol 99.9%	0.125–64
Tylosin		H_2_O	0.125–64
Lincomycin	Lincosamides		2–1024
Streptomycin	Aminoglicosides	H_2_O	2–1024
Gentamicin		BR II^a^	1–512
Neomycin		BR II^a^	1–512
Chloramphenicol	Chloramphenicol	Methanol 50%	0.5–256
Tiamulin	Semisynthetic derivative of pleuromutilin	–	0.5–256
Enrofloxacin	Fluoroquinolones	–	0.5–512
Flumequine		0.1 M NH_4_OH	2–1024

^a^BR II buffer: 16.73 g K_2_HPO_4_ and 0.523 g KH_2_PO_4_ diluted in 1000 ml of distilled H_2_O, pH 7.9

The breakpoints for testing lactobacilli are not reported in the European Committee on Antimicrobial Susceptibility Testing (EUCAST) guidelines, and the procedure proposed by the CLSI (M-45) is not adapted to the growth requirements of all *Lactobacillus* species. Hence our interpretation of the results was based on the breakpoint values suggested by FEDAP [9] for ampicillin, tetracycline, erythromycin, streptomycin, gentamicin and chloramphenicol. For doxycycline and tylosin we adopted the breakpoints of tetracycline and erythromycin, respectively. For lincomycin, tiamulin and enrofloxacin we established cut-offs based on the distribution of MICs (bimodal) and the presence or absence of resistance genes. The bacteria were considered resistant if the MIC was ≥8 μg/ml for tiamulin, ≥32 μg/ml for lincomycin, and ≥64 μg/ml for enrofloxacin. No breakpoints for neomycin and flumequine were proposed since the MICs generally showed unimodal distribution.

### Detection of resistance genes

Bacterial genomic DNA was isolated using a GeneMATRIX Bacterial & Yeast Genomic DNA Purification Kit (Eurx, Poland), following the manufacturer’s instructions with some modifications [10].

PCR reactions using gene-specific primers (Table 2) were used to detect the presence of 36 genes known to be involved in resistance to the antibiotics tested. Seven tetracycline and macrolide resistance genes (*tet* *M*, *tet* *K*, *tet* *L*, *tet* *O*, *erm* *A*, *erm* *B* and *mef* *A/E*) and five aminoglycoside resistance genes (Table 2) were detected using multiplex PCR [14, 15].

**Table 2:** Primers used for detection of selected antibiotic resistance genes

Determining resistance to	Target gene	Primer sequence (5′–3′)	Amplicon size (bp)	Annealing temperature (°C)	Reference
Tetracyclines	*tetM*	GTG GAC AAA GGT ACA ACG AG CGG TAA AGT TCG TCA CAC AC	406	60	[14]
	*tetK*	GAT CAA TTG TAG CTT TAG GTG AAG G TTT TGT TGA TTT ACC AGG TAC CAT T	155	60	
	*tetL*	TGG TGG AAT GAT AGC CCA TT CAG GAA TGA CAG CAC GCT AA	229	60	
	*tetO*	AAC TTA GGC ATT CTG GCT CAC TCC CAC TGT TCC ATA TCG TCA	515	60	
	*tetQ*	TTA TAC TTC CTC CGG CAT CG ATC GGT TCG AGA ATG TCC AC	904	55	[64]
	*tetW*	GAG AGC CTG CTA TAT GCC AGC GGG CGT ATC CAC AAT GTT AAC	168	64	[65]
Macrolides	*ermA*	CCC GAA AAA TAC GCA AAA TTT CAT CCC TGT TTA CCC ATT TAT AAA CG	590	60	[14]
	*ermB*	TGG TAT TCC AAA TGC GTA ATG CTG TGG TAT GGC GGG TAA GT	745	60	
	*ermC*	AAT CGT CAA TTC CTG CAT GT TAATCGTGGAATACGGGTTTG	299	58	[31]
	*ermT*	TAT TAT TGA GAT TGG TTC AGG G GGA TGA AAG TAT TCT CTA GGG ATT T	395	55	[33]
	*msrA/B*	GCA AAT GGT GTA GGT AAG ACA ACT ATC ATG TGA TGT AAA CAA AAT	399	54	[66]
	*msrC*	AAG GAA TCC TTC TCT CTC CG GTA AAC AAA ATC GTT CCC G	343	55	[67]
	*mefA/E*	CAA TAT GGG CAG GGC AAG AAG CTG TTC CAA TGC TAC GC	317	60	[14]
	*lnuA*	GGT GGC TGG GGG GTA GAT GTA TTA ACT GG GCT TCT TTT GAA ATA CAT GGT ATT TTT CGA TC	323	61	[32]
Aminoglicosides	*aac(6’)*-*Ie*-*aph(2*″*)*-*Ia*	CAG AGC CTT GGG AAG ATG AAG CCT CGT GTA ATT CAT GTT CTG GC	348	57	[15]
	*aph3IIIa*	GGC TAA AAT GAG AAT ATC ACC GG CTT TAA AAA ATC ATA CAG CTC GCG	523		
	*ant(4’)*-*Ia*	CAA ACT GCT AAA TCG GTA GAA GCC GGA AAG TTG ACC AGA CAT TAC GAA CT	294		
	*aph(2*″*)*-*Ic*	CCA CAA TGA TAA TGA CTC AGT TCC C CCA CAG CTT CCG ATA GCA AGA G	444		
	*aph(2*″*)*-*Id*	GTG GTT TTT ACA GGA ATG CCA TC CCC TCT TCA TAC CAA TCC ATA TAA CC	641		
	*ant(6)*-*Ia*	CGG GAG AAT GGG AGA CTT TG CTG TGG CTC CAC AAT CTG AT	563	56	[54]
	*aac(6’)*-*Ii*	TGGCCGGAAGAATATGGAGA GCATTTGGTAAGACACCTACG	410	55	
	*aadA*	ATC CTT CGG CGC GAT TTT G GCA GCG CAA TGA CAT TCT TG	282	55	[47]
	*aadE*	ATG GAA TTA TTC CCA CCT GA TCA AAA CCC CTA TTA AAG CC	1100	51	[51]
Chloramphenicol	*cat*	TAA GGT TAT TGG GAT AAG TTA GCA TGR TAA CCA TCA CAW AC	~300	54	[18]
Tiamulin	*cfr*	TGA AGT ATA AAG CAG GTT GGG AGT CA ACC ATA TAA TTG ACC ACA AGC AGC	746	58	[68]
	*lsaE*	TGT CAA ATG GTG AGC AAA CG TGT AAA ACG GCT TCC TGA TG	496	54	[69]
	*lsaC*	GGC TAT GTA AAA CCT GTA TTT G ACT GAC AAT TTT TCT TCC GT	429	50	[70]
	*vgaA*	AGT GGT GGT GAA GTA ACA CG CTC TTG TTC TAA TTC TTC CG	1287	53	[70]
	*vgaAv*	CTC CGT GTT GAA GAT GTT TCG GGA TTC AAA CGC CTC TAT AGC C	459	56	[71]
Penicillins	*blaZ*	ACT TCA ACA CCT GCT GCT TTC TAG GTT CAG ATT GGC CCT TAG	240	60	[72]
	*mecA*	AGT TCT GCA GTA CCG GAT TTG C AAA ATC GAT GGT AAA GGT TGG C	533	55	[73]
	*pbp5*	AAC AAA ATG ACA AAC GGG TAT CCT TGG TTA TCA GGG	779	52	[74]
Many antibiotics	*acrA*	CTC TCA GGC AGC TTA GCC CTA A TGC AGA GGT TCA GTT TTG ACT GTT	107	58	[58]
	*mdeA*	CTT TCA GGT TAC CTT GTT GAA TAT TTA AAC ATC AAT AGG TAC TTT AAT TGT AGT TCC AAC	180	56	[75]
	*norA*	TTT GTT TTC AGT GTC AGA ATT TAT GTT TG GGC TTG GTG AAA TAT CAG CTA TTA AAC	140	56	
	*mepA*	ATG GTA TAG GTT TCT TGT TTA CTG GTA TG AAT GAT AAT TGC ACC TTG TAA AAT GGC	150	57	

The reaction mixture composition (25 μl) for detection of other (single) resistance genes was as follows: 2.5 μl PCR buffer (200 mM Tris–HCl, pH 8.8, 100 mM KCl, 100 mM (NH_4_)_2_SO_4_, 1.0% Triton X-100), 1.5 μl 8 mM deoxynucleoside triphosphates (dNTPs, Blirt, Poland), 1 μl of each of two primers (10 pmol/μl, Genomed, Poland), 0.15 μl Dream Taq DNA polymerase (5 U/ml, Thermo Scientific), 1 μl template DNA (~20 ng) and 17.8 μl deionized water (Sigma, Poland).

All PCR reactions were performed in an Eppendorf Mastercycler using the following temperature program: initial denaturation at 94 °C for 5 min, 30 cycles of 94 °C for 45 s, 50–64 °C (according to the annealing temperature for the individual primers; Table 2) for 45 s, 72 °C for 75 s and a final extension step at 72 °C for 8 min. PCR products (8 μl) were separated by electrophoresis (100 V) on 2% agarose gels and visualized by ethidium bromide staining.

### Nitrocefin test

Production of β-lactamase was tested by streaking the bacterial colonies onto nitrocefin strips (DIAGNOSTICS Inc., Slovak Republic). The lactobacilli used in the test had been grown overnight on MRSA agar around a ampicillin disc (induction of production of β-lactamases). A change in colour from light yellow to red within 10 min at room temperature was identified as hydrolysis of the β-lactam antibiotic by the induced β-lactamase. As a positive control we used three wild *E. coli* isolates containing the *bla* _TEM-1_ gene (detection of *bla*_TEM-1_ was performed according to Van et al. [16]).

## Results

### Antimicrobial susceptibility testing

The MIC of 13 antibiotic agents was analysed for 88 *Lactobacillus* strains isolated from chickens. The MIC range was ≤0.25 to >64 μg/ml for ampicillin, ≤1–512 μg/ml for tetracyclines, ≤0.25 to >64 μg/ml for macrolides, ≤2 to >1024 μg/ml for lincomycin, ≤1 to >1024 μg/ml for aminoglycosides, 1 to 64 μg/ml for chloramphenicol, ≤0.5 to >256 μg/ml for tiamulin, and 2 to 512 μg/ml for fluoroquinolones (Table 3).

**Table 3:**
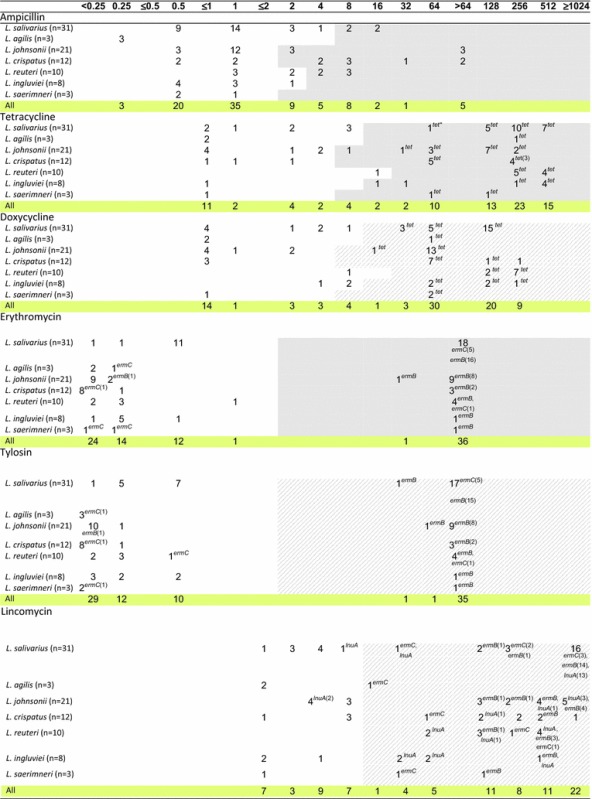

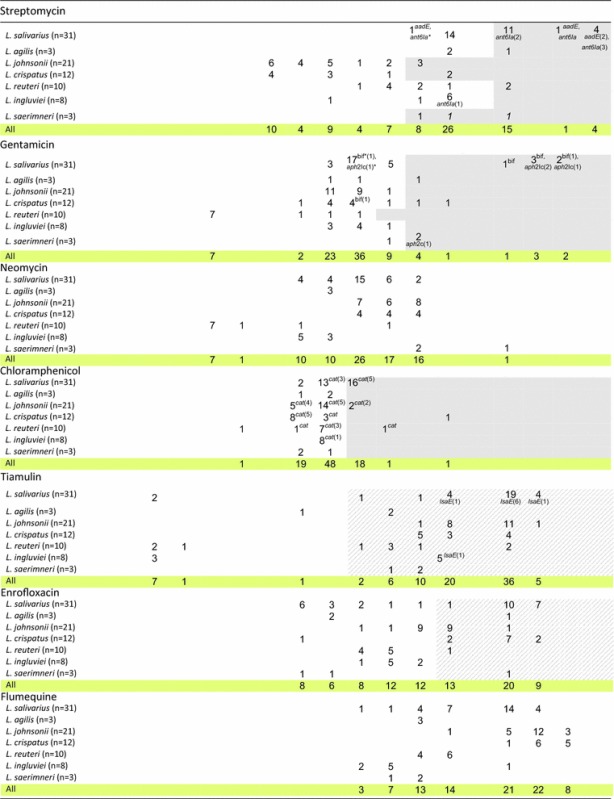
Distribution of MICs of antibiotics among various *Lactobacillus* species of chicken origin

Fragments highlighted in grey indicate MIC cut-off values (μg/ml) proposed by the EFSA [9], and strips highlighted in grey (for doxycycline, tylosin, lincomycin, tiamulin and enrofloxacin) indicate MIC cut-off values proposed by us. The number of strains carrying the gene in question is given in brackets after the name of the gene. The absence of any number following the name of the gene means that all isolates contain the gene; tet*= *tetM* or *tetL *or *tetW*; bif*= *aac(6')-Ie-aph(2")-Ia *; ant6Ia*= *ant(6)-Ia*; aph2Ic*= *aph(2”)-Ic*

According to the established criteria, 90% of isolates were resistant to tiamulin, 74% to tetracycline, 72% to doxycycline, 70% to lincomycin, 48% to enrofloxacin, 42% to erythromycin and tylosin, 31% to streptomycin, 26% to ampicillin, 23% to chloramphenicol, and 12.5% to gentamicin (Table 4). As many as 98% of *Lactobacillus* isolates showed resistance to at least one antimicrobial agent. Multiple-drug resistance was observed for 79.5% of lactobacilli (Table 4). Seven isolates (8%; *L. salivarius* 8a, 9b, *L. johnsonii* 3c, 4b, 8f, *L. agilis* 6 days and 8 h) were resistant to only one antibiotic, and in all cases it was tiamulin (MIC ≥8 µg/ml). Only two *L. ingluviei* isolates (Ch9e, Ch43d) showed susceptibility to all the drugs tested (the analysis did not include neomycin or flumequine).

**Table 4:** Number of resistant *Lactobacillus* strains determined on the basis of MIC breakpoints established for the antibiotics

	Number of strains													
	Do not showing any resistance	Susceptible to one drug only	With multidrug resistance^a^	Displayed resistance against										
				Ampicillin (MIC ≥2 or 4 or 8 μg/ml)	Tetracyclines (MIC ≥8 or 16 or 32 μg/ml)	Macrolides (MIC ≥2 μg/ml)	Lincomycin (MIC ≥16 μg/ml)	Streptomycin (MIC ≥32 or 128 μg/ml)	Gentamicin (MIC ≥16 or 32 μg/ml)	Chloramphenicol (MIC ≥8 μg/ml)	Tiamulin (MIC ≥8 μg/ml)	Enrofloxacin (MIC ≥64 μg/ml)	Macrolides and lincomycin	Streptomycin and gentamicin
*L. salivarius* (n = 31)	0 (0%)	2 (6%)	27 (87%)	4 (13%)	23 (74%)	18 (58%)	22 (71%)	16 (52%)	6 (19%)	16 (52%)	29 (93.5%)	18 (58%)	18 (58%)	4 (13%)
*L. agilis* (n = 3)	0 (0%)	2 (67%)	1 (33%)	0 (0%)	1 (33%)	0 (0%)	1 (33%)	1 (33%)	1 (33%)	0 (0%)	2 (67%)	1 (33%)	0 (0%)	1 (33%)
*L. johnsoni* (n = 21)	0 (0%)	3 (14%)	16 (76%)	6 (28%)	14 (67%)	10 (48%)	14 (67%)	3 (14%)	0 (0%)	2 (9.5%)	21 (100%)	10 (48%)	10 (48%)	0 (0%)
*L. crispatus* (n = 12)	0 (0%)	0 (0%)	10 (83%)	8 (67%)	9 (75%)	3 (25%)	8 (67%)	2 (17%)	2 (17%)	1 (8%)	12 (100%)	11 (92%)	3 (25%)	1 (8.3%)
*L. reuteri* (n = 10)	0 (0%)	0 (0%)	9 (90%)	5 (50%)	9 (90%)	4 (40%)	10 (100%)	2 (20%)	0 (0%)	1 (10%)	7 (70%)	1 (10%)	4 (40%)	0 (0%)
*L. ingluviei* (n = 8)	2 (25%)	0 (0%)	5 (62.5%)	0 (0%)	7 (87%)	1 (12%)	5 (62%)	0 (0%)	0 (0%)	0 (0%)	5 (62.5%)	0 (0%)	1 (12%)	0 (0%)
*L. saerimneri* (n = 3)	0 (0%)	0 (0%)	2 (67%)	0 (0%)	2 (67%)	1 (33%)	2 (67%)	3 (100%)	2 (67%)	0 (0%)	3 (100%)	1 (33%)	1 (33%)	2 (67%)
Total: 88	2 (2%)	7 (8%)	70 (79.5%)	23 (26%)	65 (74%)	37 (42%)	62 (70%)	27 (31%)	11 (12.5%)	20 (23%)	79 (90%)	42 (48%)	37 (42%)	8 (9%)

^a^Resistant to at least 3 groups of antimicrobial agents

The ampicillin-resistant isolates (23) belonged to different *Lactobacillus* species. The highest MIC values, i.e. >64 μg/ml, were observed for 5 isolates of *L. johnsonii* and *L. crispatus*, with clear bimodal distribution of MICs noted only for *L. johnsonii* isolates. In the case of *L. crispatus* we noted three MIC ranges −05 to 1 μg/ml, 4 to 8 μg/ml and 32 to >64 μg/ml, which could indicate the presence of sensitive, intermediate and resistant strains (Table 3).

Clear bimodal distribution of MICs indicative of acquired resistance was observed for macrolides, where the bimodal distribution obtained indicated two subpopulations: one susceptible, with low MICs (≤0.25–1 µg/ml), and the other resistant, with high MICs (32 to >64 µg/ml). Similar clear bimodal distribution of MICs was noted for tiamulin for four species—*L. salivarius*, *L. agilis*, *L. reuteri* and *L. ingluviei*, and the MIC of 8 isolates was only ≤0.5 μg/ml (Table 3).

In the case of tetracyclines, lincomycin and enrofloxacin, bimodal distribution of MICs was observed for most *Lactobacillus* species, but was not as clearly bimodal as for macrolides (Table 3).

In the case of aminoglycosides, chloramphenicol and flumequine, the MIC values were distributed unimodally for most *Lactobacillus* species. Clear bimodal distribution was observed only in the case of gentamicin for *L. salivarius*, streptomycin and flumequine for *L. ingluviei*, neomycin for *L. reuteri*, and chloramphenicol for *L. crispatus*. Large differences were noted between *Lactobacillus* species in terms of their susceptibility to streptomycin. High MIC values ≥32 µg/ml characterised *L. salivarius* and *L. saerimneri* isolates, while for *L. johnsonii* and *L. crispatus* MICs were in the range of ≤2–64 µg/ml. The high sensitivity of most *L. reuteri* isolates to gentamicin and neomycin (MIC ≤1 µg/ml) should also be highlighted (Table 3).

Lactobacilli exhibited cross-resistance to antibiotics belonging to the same chemical groups; 74% of strains were simultaneously resistant to tetracycline and doxycycline, 42% showed cross-resistance between erythromycin and tylosin, and for 42% (37) of lactobacilli cross-resistance was observed between macrolides and lincosamides. Simultaneous resistance to streptomycin and gentamicin was recorded for 9% of isolates (Table 4).

### Detection of antibiotic resistance genes

To explain the mechanism responsible for the resistance phenotypes observed, all *Lactobacillus* isolates were screened by PCR for the presence of genetic determinants of resistance to the antibiotic agents tested.

Thirteen of the 36 investigated resistance genes were detected in the lactobacilli. All isolates contained efflux pump gene *acr*A. We found *tet* genes in 61 of 65 isolates showing a tetracycline resistance phenotype (MICs ≥8 or ≥16 or ≥32 μg/ml). The most frequently occurring *tet* gene was *tet*W, which was observed in 45% of isolates, followed by *tet*M (26%) and *tet*L (24%). The *tet*W gene was noted in *L. reuteri* strains (90% of isolates contained *tet*W), *L. ingluviei* (75%), *L. saerimneri* (67%), *L. johnsonii* (62%) and *L. crispatus* (58%), but not in the *L. salivarius* isolates. The presence of the *tet*M and *tet*L genes was characteristic for *L. salivarius*, as 68% of isolates contained *tet*M, 61% carried *tet*L and 58% had both genes (Tables 3, 5). Co-occurrence of the *tet*L, *tet*M and *tet*W genes was observed in only one isolate.

**Table 5:** Number of *Lactobacillus* strains carrying resistance genes

Resistant gene	*tetL*	*tetM*	*tetW*	*ermB*	*ermC*	*LnuA*	*tetL* + *tetM*	*tetL* + *tetM* + *ermB*	*tetW* + *ermB*	*ermB* + *ermC*	*ermB* + *lnuA*	*cat*	*ant(6)*-*Ia*	*aadE*	*ant(6)*-*Ia* + *aadE*	*aac(6’)*-*Ie*-*aph(2’)*-*Ia*	*aph(2*″*)*-*Ic*	*lsaE*	*lsaE* + *ant(6)*-*Ia*	*acrA*
*L. salivarius* (n = 31)	19 (61%)	21 (68%)	0	16 (52%)	6 (19%)	15 (48%)	18 (58%)	14 (45%)	0	2 (1%)	12 (39%)	8 (26%)	8 (26%)	4 (13%)	4 (13%)	6 (19%)	4 (13%)	8 (26%)	7 (22%)	31 (100%)
*L. johnsonii* (n = 21)	0	0	13 (62%)	10	0	6 (3%)	0	0	7 (3%)	0	4 (2%)	11 (48%)	0	0	0	0	0	0	0	21 (100%)
*L. crispatus* (n = 12)	1 (8%)	1 (8%)	7 (58%)	2 (17%)	1 (8%)	1 (8%)	1 (8%)	1 (8%)	1 (8%)	0	0	8 (7%)	0	0	0	1 (8%)	0	0	0	12 (100%)
*L. reuteri* (n = 10)	0	0	9 (90%)	4 (40%)	2 (20%)	7 (70%)	0	0	4 (40%)	1 (1%)	3 (3%)	5 (50%)	0	0	0	0	0	0	0	10 (100%)
*L. ingluviei* (n = 8)	0	0	6 (75%)	1 (12%)	0	5 (62%)	0	0	1 (12%)	0	1 (12%)	1 (12%)	1 (12%)	0	0	0	0	2 (25%)	1 (12%)	8 (100%)
*L. agilis* (n = 3)	1 (33%)	1 (33%)	0	0	1 (33%)	0	1 (33%)	0	0	0	*–*	0	0	0	0	0	0	0	0	3 (100%)
*L. saerimneri* (n = 3)	0	0	2 (67%)	1 (33%)	1 (33%)	0	*0*	0	1 (33%)	0	0	0	0	0	0	0	1 (33%)	0	0	3 (100%)
Total: 88	21 (24%)	23 (26%)	37 (42%)	34 (39%)	11 (12%)	34 (39%)	20 (23%)	15 (17%)	14 (16%)	3 (3%)	20 (23%)	33 (37.5%)	9 (10%)	4 (4.5%)	4 (4.5%)	7 (8%)	5 (6%)	10 (11%)	8 (9%)	88 (100%)

Non of the isolate contained the resistance genes: *tet* *K*, *tet* *O*, *tet* *Q*, *erm* *A*, *erm* *T*, *mef* *A/E*, *msr* *A/B*, *msr* *C*, *bla* *Z*, *mec* *A*, *pbp* *5*, *aad* *A*, *aph(3’)*-*IIIa*, *aph* *(2″)-Id*, *ant(4’)*-*Ia, aac(6’)*-*Ii, cfr, lsaC, vgaA, vgaAv, mdeA, norA, mepA*

The genes *ermB*, *ermC* (coding methylases) and *lnuA* (coding lincosamide *O*-nucleotidyltransferase), associated with resistance to macrolides and lincosamides, were present in 39, 12 and 39% of isolates, respectively. The *ermB* gene occurred in all isolates resistant to erythromycin and tylosin (37 isolates, MIC ≥32 μg/ml) and in 34 of 62 isolates considered resistant to lincomycin (MIC≥16 μg/ml). Thirty-three isolates containing *ermB* showed cross-resistance to macrolides and lincosamides, and one isolate (*L. johnsonii*) carrying *ermB* was susceptible to macrolides but resistant to lincomycin. The *ermB* gene was mainly present in strains of *L. salivarius* (53%) and *L. reuteri* (40%). The *ermC* gene was detected in 11 isolates (12%) of various *Lactobacillus* species. All the *ermC*-carrying isolates showed phenotypic resistance to lincomycin, but 6 of them were susceptible to macrolides. The *lnuA* gene was detected in 34 strains, including 31 of 62 strains that were resistant to lincomycin and 3 that were susceptible to it (Tables 3, 5).

Genes determining resistance to aminoglycoside antibiotics were detected in 15 isolates (17%), belonging mainly to the genus *L. salivarius*. The most common were *ant(6)*-*Ia* (10% of isolates) and *aac(6*′*)*-*Ie*-*aph(2*′*)*-*Ia* (8%), followed by *aph(2*″*)*-*Ic* (6%) and *aadE* (4.5%). These genes occurred in both phenotypically resistant and sensitive isolates belonging to the species *L. salivarius*, *L. crispatus*, *L. ingluviei* and *L. saerimneri*. The gene *aac*(*6*′*)*-*Ie*-*aph*(*2″)*-*Ia* encoding bifunctional aminoglycoside-modifying enzyme, which confers high-level resistance to gentamicin [17], was present in 7 isolates (6 isolates of *L. salivarius* and one of *L. crispatus*), 4 of which exhibited phenotypic resistance to gentamicin. The gene *aph(2*″*)*-*Ic* was detected in 3 isolates of *L. salivarius* and one *L. saerimneri* isolate showing resistance to gentamicin and in one gentamicin-susceptible isolate of *L. salivarius*. The presence of *ant(6)*-*Ia* and *aadE* genes encoding ANT(6) adenyltransferases, which determine resistance to streptomycin [17], was noted in 11 isolates (9 of *L. salivarius*, one *L. reuteri* and one *L. ingluviei*). The *ant(6)*-*Ia* gene was present in 7 of 24 streptomycin-resistant isolates and in 2 isolates considered to be streptomycin-susceptible. Four *L. salivarius* isolates, including 3 streptomycin-resistant and one streptomycin-susceptible, contained both the *ant(6)*-*Ia* and *aadE* genes (Tables 3, 5).

The *cat* gene encoding chloramphenicol acetyltransferase, which converts chloramphenicol to inactive diacetyl chloramphenicol [18], was present in 32 isolates (36%), including 8 of 20 found to be resistant to chloramphenicol (MIC ≤8 μg/ml) (Tables 3, 5).

In PCR using *lsaE*-specific primers, we obtained a single product for 20 isolates, but with varying product size—496 bp for 8 isolates of *L. salivarius* and 2 of *L. ingluviei*, and 570 bp for 10 isolates of *L. johnsonii*. Therefore we sequenced representative PCR products of both sizes. Only the PCR product of 496 bp (GenBank Accession No. KY924692) showed 99% similarity (NCBI BLAST algorithm) to the the *lsaE* gene sequence of *Enterococcus faecalis* deposited in GenBank (Accession Nos. KX156279.1, KX156278.1 and NG_047935.1). The *lsaE* gene was present in 10 of 79 isolates that were considered phenotypically resistant to tiamulin (MIC ≥8 μg/ml), and 8 of these *lsaE*-positive isolates also contained the *aadE* or *ant(6)*-*Ia* gene conferring resistance to streptomycin (Tables 3, 5).

Some *Lactobacillus* strains simultaneously contained genes responsible for resistance to different antibiotics (Table 5).

None of the *Lactobacillus* isolates tested contained the *tetK*, *tetO*, *tetQ*, *ermA*, *ermT*, *mefA/E, msrA/B*, *msrC*, *blaZ*, *mecA*, *pbp5*, *aadA*, *aph(3*′*)*-*IIIa*, *aph(2*″*)*-*Id*, *ant(4*′*)*-*Ia*, *aac(6*′*)*-*Ii*, *cfr*, *lsaC*, *vgaA*, *vgaAv*, *mdeA*, *norA* or *mepA* genes.

### Nitrocefin test

The result of the nitrocefin test, which is considered a highly sensitive method for detection of the bacterial β-lactamase enzyme [19], was negative for all ampicillin-resistant *Lactobacillus* isolates tested.

### Distribution of resistant isolates on farms

In this work, we observed considerable variation in the percentage of resistant isolates among lactobacilli collected from individual farms. The range of ampicillin resistance was 9–62.5%, tetracyclines 14–100%, lincomycin 8–100%, macrolides 0–92%, streptomycin 0–54%, gentamicin 0–23.5%, chloramphenicol 0–53%, tiamulin 75–100% and enrofloxacin 25–82% (Table 6).

**Table 6:** Percentage of resistant lactobacilli isolated from individual chicken farms

Farm Age of birds/no. of *Lactobacillus* isolates	Broiler chickens						Green-legged Partridge hens	
	I 45 days/11 (%)	II 49 days/13 (%)	III 17 days/17 (%)	IV 42 days/7 (%)	V 40 days/8 (%)	VI 48 days/12 (%)	VII 2 days/7 (%)	VIII 49 days/13 (%)
Ampicillin	9	15	29	43	62.5	25	28.5	15
Tetracyclines	100	92	94	86	100	58	14	31
Lincomycin	91	100	100	86	62.5	50	57	8
Macrolides	54.5	92	70.5	43	25	8	0	8
Streptomycin	45	54	29	0	0	50	28.5	15
Gentamicin	9	23	23.5	0	0	17	14	0
Chloramphenicol	9	31	53	0	12.5	25	0	15
Tiamulin	82	92	88	100	75	100	86	92
Enrofloxacin	27	61.5	82	57	25	42	28.5	31
Isolates without any resistance	0	0	0	0	12.5	0	0	8
Resistance to one drug only	0	0	0	0	0	0	43	31
Multidrug resistant isolates	100	100	100	100	75	67	57	31

All isolates from farm VII were sensitive to macrolides, and isolates from farms IV and VII—to chloramphenicol. No aminoglycoside resistance was observed on farms IV and V. Lactobacilli from farm VIII were characterized by a low percentage of isolates resistant to lincomycin (8%).

The lowest percentage of multiresistant isolates (31–57%) and tetracycline resistant strains (14–31%) was recorded on two farms raising Green-legged Partridge hens. Among 20 *Lactobacillus* isolates derived from these farms, one strain of *L. ingluviei* (Ch9e) did not show resistance to any antibiotic and 7 isolates showed phenotypic resistance to only one antibiotic (tiamulin); none of these 8 isolates contained any resistance genes, except *acrA*.

## Discussion

In this study we assayed 88 *Lactobacillus* isolates of chicken origin with regard to their susceptibility to 13 antibacterial agents. We found high prevalence of resistance to tiamulin (90% resistant isolates), tetracyclines (74%), and lincosamides (70%), and moderately high frequency of resistance to enrofloxacin (48%), macrolides (42%), aminoglycosides (12.5–31%), ampicillin (26% resistant isolates) and chloramphenicol (23%).

Tiamulin, doxycycline, chlortetracycline, oxytetracycline, erythromycin, tylosin, lincomycin and enrofloxacin are currently approved for treatment of poultry diseases in Poland, and in the past some of these antibiotics were commonly used as feed additives for chickens. The intensive use and misuse of antibiotics in animal husbandry are unquestionably the major forces contributing to the development of resistance in bacteria, both pathogenic and commensal [20].

In this work we have presented the first reliable data on the sensitivity of lactobacilli to tiamulin. According to the proposed criteria, most isolates (90%) were resistant to tiamulin (MIC ≥8 μg/ml) and the most commonly observed MIC value was 64–128 μg/ml (for 64% of isolates). For *L. salivarius*, *L. agilis*, *L. reuteri* and *L. ingluviei*, we observed bimodal distribution of MIC values, with the MIC of 9% isolates as low as ≤0.5 μg/ml. MIC values for tiamulin determined by Karpetkov et al. [21] for 3 isolates of *Lactobacillus* (*L. acidophilus*, *L. helveticus and L. bulgaricus*) were in the range of 0.5–1 μg/ml, while the sensitivity of other Gram-positive bacteria to tiamulin is varied. Callens et al. [22] showed that the MIC for *Streptococcus suis* (332 isolates tested) from pigs ranged from 0.03 to 128 μg/ml, and MIC = 4 μg/ml was established as an epidemiological cut-off value. Jones et al. [23] showed that MIC90 was >32 μg/ml for enterococci (71 isolates tested), and 2 μg/ml for *S. aureus* (150 isolates).

The observed phenotypic resistance of lactobacilli to tiamulin may be due to mutation in the 23S rRNA or in the *rplC* genes encoding ribosomal proteins. The presence of multidrug efflux pumps (*vga* and *lsa*) is also possible [24]. None of the isolates contained the *cfr* gene coding for RNA methyltransferase or the *vgaA*, *vgaAv* and *lsaC* genes encoding ABC transporters that confer combined resistance to pleuromutilins, lincosamides and streptogramin A antibiotics (PLS_A_) in Gram-positive bacteria [24]. However, in 10 isolates we detected the *lsaE* gene, which has previously been identified in staphylococci, enterococci (Europe, Asia) and *Streptococcus agalactiae* (South America) [25]. Eight *lasE*-positive isolates simultaneously contained the *aadE* or *ant(6)*-*Ia* gene conferring resistance to streptomycin. This observation is in line with findings by Si et al. [25], who demonstrated the presence of the *lsaE* gene within plasmid or chromosomal clusters comprising several resistance genes, including *aadE*.

High prevalence of tetracycline resistance (74% of isolates) in the lactobacilli tested is in line with observations by Cauwerts et al. [3], who found that over 78% of lactobacilli isolated from Belgian broiler farms were resistant to tetracycline, with a particularly high rate of resistance observed among strains of *L. reuteri*, *L. gallinarum*, *L. crispatus* and *L. salivarius*. Also, Vieira De Souza et al. [26] reported a high incidence of tetracycline resistance (MIC ≥128 μg/ml) among lactobacilli isolated from the GIT of free-range broiler chickens when resistance was determined by the microplate method using MRS broth. A lower incidence of resistance to tetracyclines (28%) and macrolides (29%) in faecal chicken lactobacilli was noted by Kmet and Piatnicova [27].

Acquired tetracycline resistance in bacteria is determined mainly by *tet* genes, which code for energy-dependent efflux proteins (e.g., *tetK* and *tetL*) or ribosomal protection proteins (e.g., *tetM*, *tetO*, *tetQ or tetW*) [28, 29]. We showed the presence of *tetL*, *tetM* and *tetW* in the *Lactobacillus* isolates with phenotypic tetracycline-resistance. The occurrence of these genes, as well as *tetK* and *tet*Z, in chicken lactobacilli (76% of strains tested) was also observed by Cauwerts et al. [3]. However, in contrast to our results, these authors more frequently detected *tetL* and *tetM* than *tetW*. In a study by Chang et al. [30], 100% of *Lactobacillus* strains isolated from swine intestines (in Taiwan) were resistant to tetracyclines; among 5 *tet* genes detected the most predominant was *tetW* (in 82% strains), followed by *tetM* (22.5%), *L* (14.4%), *K* (8.1%) and *Q* (0.9%). The *tetW* and *tetM* genes have also been found in *Lactobacillus* strains derived from humans, probiotics and food products Klare et al. [31]. Our finding that the *tetW* gene is characteristic for *L. crispatus*, *L. johnsonii* and *L. reuteri* is consistent with the observations of other authors [3, 32, 33].

High rates of resistance to macrolides and lincosamides among chicken lactobacilli have previously been reported by Cauwerts et al. [4], who demonstrated that 78% of *Lactobacillus* strains (belonging to 5 species) from Belgian broiler farms displayed resistance to erythromycin and tylosin (MIC ≥16 μg/ml) and 87% were resistant to lincomycin (MIC ≥64 μg/ml). Chin et al. [34] found that 58% of lactobacilli isolated from the GIT of chickens exhibited a high degree of resistance to erythromycin (MIC ≥200 μg/ml). A clear bimodal distribution of MICs for erythromycin and tylosin, indicative of acquired resistance, has also been reported by other authors in MLS-resistant lactobacilli derived from various sources [4, 12, 33, 35].

We have shown that the vast majority of *Lactobacillus* strains characterized by phenotypic resistance to macrolides and/or lincosamides contained resistance-related genes. PCR detection of methylase genes (*erm*), efflux genes (*mef* and *msr*) and the lincosamide *O*-nucleotidyltransferase gene (*lnuA)* confirmed a high incidence of *ermB* (in 39% of isolates) and *lnuA* (39%) in chicken lactobacilli, while *ermC* occurred less frequently (12%). Our results are consistent with those of Cauwerts et al. [4], who found that carriage of the *erm*B gene always concurred with phenotypic resistance to macrolides and lincosamides in chicken lactobacilli. The same authors reported the occasional occurrence of the *lnuA*, *mefA* and *ermC* genes. The *ermB* gene is also widespread in other poultry-derived LAB [36, 37]. Four of 11 *ermC*-positive isolates were susceptible to macrolides and at the same time displayed low resistance to lincomycin (MIC 16–64 μg/ml). Our results are consistent with those of Cauwerts et al. [4], who showed that chicken *Lactobacillus* strains may carry the *ermC* gene without exhibiting phenotypic macrolide or lincosamide (MIC ≥64 μg/ml) resistance. Note, however, that according to the cut-offs adopted by Cauwerts et al. [4], three of our strains carrying the *ermC* gene would be considered susceptible to both macrolides and lincosamides.

In this work, the *lnuA* gene encoding a transferase inducing inactivation of lincosamides was detected mainly in *L. reuteri* and *L. ingluviei* strains. The presence of *lnuA* in *L. reuteri* has previously been reported by Cauwerts et al. [4] (in chicken lactobacilli) and by Kastner et al. [32] (in the probiotic strain *L. reuteri* ATCC 55730).

Resistance to tetracyclines and macrolides and the *tet* and *erm* genes also occurs among *Lactobacillus* and *Lactococcus* strains isolated from various food sources, including poultry meat products [38, 39].

The incidence of ampicillin resistance (26% isolates) recorded in this study is higher than that observed by other researchers working on poultry LAB over the past 15 years. Kmet and Piatnicova [27] showed that 100% of *Lactobacillus* strains from cloacal swabs of broiler chickens raised on farms in Slovakia were susceptible to penicillin and ampicillin. High sensitivity of LAB isolated from Malaysian broiler chickens to β-lactams (penicillin, ampicillin and amoxicillin) has also been reported by Shazali et al. [40], and Lonkar et al. [41] noted only sporadic β-lactam resistance among poultry lactobacilli (≤ 3.5%). Low ampicillin MICs (MIC ≤4 μg/ml) have also been demonstrated for goose-derived lactobacilli [11]. We were unable to explain the resistance mechanism of *Lactobacillus* isolates against ampicillin, but we ruled out the involvement of β-lactamases. This is consistent with other studies demonstrating the absence of the *bla*Z gene in lactobacilli despite their phenotypic resistance to β-lactam antibiotics [12, 18].

In our study, 23% of isolates showed resistance to chloramphenicol, but for most of them the MICs were 8 μg/ml, while the established cut-off value is 4 μg/ml. A high MIC value, 64 μg/ml, was recorded for only one isolate. The *cat* gene encoding chloramphenicol acetyltransferase was detected in 37.5% of isolates, both phenotypically resistant and susceptible. A similar range of MIC values for chloramphenicol, i.e. 1–8 μg/ml for most lactobacilli tested, was observed by Mayrhofer et al. [42] and by Danielsen and Wind [43], while high MIC values ≥32 μg/ml have been noted only occasionally [12, 43]. Hummel et al. [18] demonstrated that the *cat* gene can be present in chloramphenicol-susceptible lactobacillus isolates, and furthermore that the *cat* gene in these strains was not expressed (RNA level) in either inducing or non-inducing conditions. The authors speculated that a mutation in the regulatory region may be responsible for the inhibition of *cat* expression in phenotypically susceptible isolates.

The frequency of resistance to aminoglycoside antibiotics among the *Lactobacillus* isolates was in a range of 12.5–31%, with *L. salivarius* dominant among resistant isolates. High MIC values, i.e. ≥128 μg/ml for streptomycin, gentamicin and neomycin, were reported for 18, 7 and 1% of isolates, respectively. Similarly, high-level resistance (MIC ≥128 μg/ml) to streptomycin and gentamicin were reported by Danielsen and Wind [43] in 61 and 3%, respectively, of *Lactobacillus* isolates of different species. Greater susceptibility of lactobacilli to aminoglycosides was observed by Korhonen [44] in bovine isolates, with MIC ranges of 0.25–8 μg/ml for gentamicin and 0.25–32 μg/ml for neomycin, while in the case of streptomycin an MIC of 2–32 μg/ml was recorded for 98% of isolates and MIC = 128 μg/ml for only one *L. salivarius* strain.

We were not able to establish the resistance status of all phenotypically resistant isolates, but in some of them we detected genes encoding aminoglycoside-modifying enzymes. Enzymatic modification is the most common type of aminoglycoside resistance, and the modifying enzymes are divided into three groups: N-acetyltransferases (AAC), O-adenyltransferases (ANT, e.g., the ANT(6) group encoded by *ant(6)*-*Ia*, *ant(6)* and *aadE* and the ANT(3″) group encoded by *aadA* genes) and O-phosphoryltransferases (APH; encoded by *aph* genes, including *aph*(3**′** *)*-*IIIa* and *aph(6)*-*Ia*, also known as *strA* [17].

Among the lactobacilli tested we identified *ant(6)*-*Ia* (10% isolates), *aac(6*′*)*-*Ie*-*aph(2*′*)*-*Ia* (8%), *aph(2*″*)*-*Ic* (6%) and *aadE* (4.5%). Our results are largely consistent with literature data, which indicate that the most frequently detected aminoglycoside resistance genes in LAB are *aac(6* **′** *)*-*Ie*-*aph(2*″*)*-*Ia*, *ant(6)*-*Ia*, *ant(6)*, *aac(6* **′** *)*-*Ii*, *aph(2*″*)*-*Ic*, *aph(3* **′** *)*-*IIIa*, *aadA* and *aadE* [15, 45–48]. Tenorio et al. [49] demonstrated the presence of the bifunctional gene *aac*(*6* **′** *)*-*Ie*-*aph(*2*”)*-*Ia* in 7 of 9 phenotypically gentamicin-resistant (MIC of ≥ 64 μg/ml) strains, including *L. salivarius*, from pigs and pets. Jaimee and Halami [50] and Rojo-Bezares et al. [48] noted the presence of *aac(6*′*)Ie*-*aph(2*″*)Ia*, *aph(3*′*)*-*IIIa*, *aad6* and *ant*(6) among *L. plantarum* isolates derived from meat products or wine. The presence of *aadE*, *aph*(*3*′*)*-*IIIa* and *aadA* in 3 of 16 tested isolates of *L. casei*, *L. paracasei* and *L. plantarum* was reported by Ouoba et al. [51]. Similarly to our results, the presence of aminoglycoside resistance genes in phenotypically sensitive lactobacillus strains has been observed by Shao et al. [47], who detected *ant(6)*, *aadE and aadA* (conferring resistance to streptomycin) in 3 *L. casei* isolates (streptomycin MIC 16–32 μg/ml) and in 5 *L. plantarum* isolates (MIC 16–512 μg/ml) from food sources. The authors stated that these eight isolates were phenotypically resistant to streptomycin, but according to breakpoints established by the EFSA (2012), *L. casei* strains are regarded as resistant if streptomycin MIC ≥128 μg/ml, while there are no established breakpoints for *L. plantarum*. Another imprecision in the publication by Shao et al. [47] is the size of the PCR product for the *aadE* gene—the amplicon size obtained using the described primers is 1100 bp, not 565 bp.

In this work we observed the co-occurrence of *ant(6)*-*Ia* and *aadE* genes in a few *L. salivarius* isolates. Both genes determine resistance to streptomycin, belong to the ANT(6) subclass [17] and some authors use their names interchangeably [52, 53]. Thus it is very likely that *ant(6)*-*Ia* and *aadE* identified in this study are the same gene detected with different primers. Sequencing of *ant(6)*-*Ia* and *aadE* amplicons revealed 97–99% homology to the sequence of *aadE* gene located on the *lsa(E)*-carrying multidrug resistance cluster of *Enterococcus faecalis* (GenBank Accession No. KX156279.1) and to *aadE* gene of *Staphylococcus aureus* (GenBank Accession No. JQ861959.1) (data not shown).

The range of MICs observed in this work for fluoroquinolones, i.e. 2–256 μg/ml for enrofloxacin and 8–512 μg/ml for flumequine, was similar to the range of MICs previously recorded for goose lactobacilli [11]. However, according to the proposed cut-off value, the rate of enrofloxacin-resistant strains (MIC ≥64 μg/ml) was higher among chicken isolates (48%) than in isolates derived from geese (23%). Other authors have reported greater susceptibility of LAB to enrofloxacin. The MIC range noted by Ishihara et al. [54] for lactobacilli isolated from dairy products was 1–8 μg/ml, and in the case of bovine lactobacilli MIC ≥64 μg/ml was noted for only 3% of isolates [44]. The presence of low MIC values for enrofloxacin, i.e. 0.5–2.0 μg/ml, was also observed by Marrow et al. [55] for enterococci isolates from free-living and captive raptors.

Among the genes coding efflux pumps we detected only *acrA*, which was present in all *Lactobacillus* isolates. High prevalence of *acrA* among LAB isolated from fermented olives has previously been noted by Casado Muñoz et al. [56]. These authors also observed a high frequency of *mepA* and *mdeA* and lower frequency of *norA*. According to some reports [57, 58], the overexpression of all these genes in *Enterobacteriaceae* is correlated with fluoroquinolone and multidrug resistance. In this work we did not evaluate the expression of the *acrA* gene, but its presence in all isolates indicates its role in cell physiology rather than its involvement in antibiotic resistance.

Resistance genes are commonly found on mobile genetic elements, such as plasmids, transposons or integrons, contributing to their widespread distribution among bacteria. Some authors have shown that LAB, including lactobacilli, may contain *tetL*-, *tetM*-, *tetW*-, *ermB*-, *ermC, lnuA*, *aac*(*6*′*)*-*Ie*-*aph(*2*”)*-*Ia*-, *aadE*-, *ant(6)*-*Ia* or *lsaE*-carrying plasmids or transposons [33, 59–63]. It has also been shown that the resistance genes *ermB* and *tetM* can be transferred between different *Lactobacillus* species, as well from lactobacilli to other LAB bacteria, including potentially pathogenic strains of *E. faecalis* [26, 60].

## Conclusions

Our work presents a comprehensive study on the antimicrobial nature of chicken lactobacilli, and includes the first report of the susceptibility of bacteria of the genus *Lactobacillus* to tiamulin. This is also the first record demonstrating the presence of the *lsaE* gene in lactobacilli, as well as some other resistance genes that have not previously been detected in the *Lactobacillus* species tested in this work.

We have shown a high frequency of resistance (≥70% resistant isolates) to tiamulin, tetracyclines and lincosamides among *Lactobacillus* isolates of chicken origin, and the prevalence of resistance to the other drugs tested ranged from 12.5 to 48%.

A major contributor to the development of antibiotic resistance in commensal microflora is their widespread use in poultry farming. Therefore it is essential to advise veterinarians and farmers to limit the use of antibiotics in chickens and to draw their attention to alternative methods of prevention and treatment.

Due to the prevalence of molecular determinants of drug resistance, chicken intestinal lactobacilli can be considered a reservoir of resistance genes. Since these genetic determinants are generally associated with mobile elements of the bacterial genome, they may be transferred to potentially pathogenic bacteria, including zoonotic agents. Thus their presence in lactobacilli may contribute to the development of opportunistic infections in poultry and may constitute a potential public health hazard.

The data derived from this study can be used as a basis for reviewing present microbiological breakpoints for categorization of susceptible and resistant strains within the genus *Lactobacillus*.
